# TraqBio - Flexible Progress Tracking for Core Unit Projects

**DOI:** 10.1371/journal.pone.0162857

**Published:** 2016-09-27

**Authors:** Gunnar Völkel, Sebastian Wiese, Karlheinz Holzmann, Johann M. Kraus, Fabian Schneider, Matthias Görlach, Hans A. Kestler

**Affiliations:** 1 Institute of Medical Systems Biology, Ulm University, D-89069 Ulm, Germany; 2 Core Unit Mass Spectrometry and Proteomics, Ulm University, D-89069 Ulm, Germany; 3 Core Facility Genomics, Ulm University, D-89069 Ulm, Germany; 4 Leibniz Institute on Aging – Fritz Lipmann Institute and FSU Jena, D-07745 Jena, Germany; Universiteit Gent, BELGIUM

## Abstract

**Motivation:**

Core service units have become an organisational hallmark in many research institutions world wide. Such service cores provide complex state-of-the-art technologies and expertise to the research community. Typically, a user delivers material or raw data to a core. The core defines work packages for ensuing analysis and returns results back to the user. This core activity can be quite complex and time consuming and usually does not communicate itself to the outside. Naturally, the user is highly interested to follow the progress of a project once handed over to the core unit. This generates a time-intensive direct communication activity back and forth. A more effective, convenient and less disruptive way to track the status of a given project by the researcher, but also by core managers, appears highly desirable. Hence, we developed a lightweight and readily implementable web application that allows efficient progress tracking of core unit projects.

**Results:**

The web application TraqBio allows for the convenient tracking of projects. Following project set-up by the core, the user receives an e-mail containing links for tracking the project status. Examples are provided for three common core units, namely genomics, proteomics, and bioinformatics units. TraqBio is a secure lightweight web application that can be either used in a standalone setup or incorporated into an existing web server infrastructure. Being accessible not only from classical desktop computers but also from mobile devices such as smartphones and tablets, TraqBio offers easy integration into every day work.

## Introduction

An essential part of every core unit management constitutes communication with the respective user about the status and progress of individual projects. Genomics or proteomics core projects may well take several months until completion and the core unit management is contacted repeatedly by the user about project progress [1]. As a core usually runs several projects in parallel, *ad hoc* answering such questions is time consuming and disruptive, as the managing core staff needs to collect the current status of the respective project. This is even more tedious if management changes occur due to holidays or other reasons. This non-productive situation is eliminated by allowing the user to keep track of the individual project steps via a web-based progress tracking system. Such a tool enables both, the user and the core management, to keep track of the progress of individual projects at the same time and, incidentally, improve the user friendliness and interactivity of any core unit to foster a co-operative and productive research environment.

**Fig 1:**
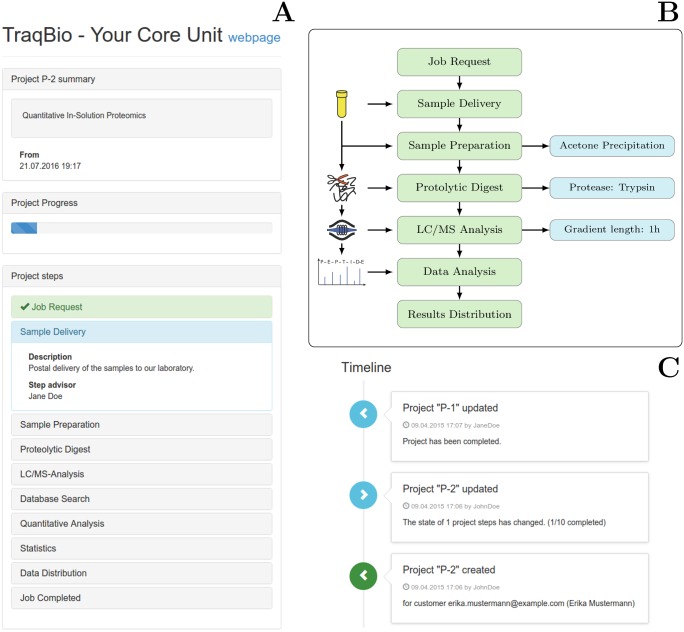
A) Tracking view of TraqBio of a typical proteomics work flow. B) Illustration of sequential work packages (green boxes) as typical for a qualitative analysis of a non-complex protein sample [1] with pictograms for the individual work packages on the left and methodological remarks, created via the free text function, on the right (blue boxes). Project steps created in the corresponding TraqBio workflow are shown in green and information related to the sample owner via the step description or the free text field is shown in blue. C) Timeline view for core unit staff indicating current status of one project created and updated and one template created from the new project.

Here, we present the web application TraqBio which allows tracking of the progress of core unit projects very much akin to tools allowing the tracking of parcel deliveries. Upon project registration in the core unit the associated user is notified by e-mail about the project’s tracking link. This serves to provide a web based user access to the project’s summary page with general information and the current status of the project (cf. Fig 1A).

TraqBio is sleek by design, intuitively usable, and a readily implementable progress tracking tool intended primarily for communicating the project status between a core and its users. It was not developed to replace a comprehensive laboratory information management system (LIMS, [2]). Main purpose of LIMS packages are laboratory internal sample tracking, auditing, documentation, quality control and reporting. Such LIMS products are available commercially and open-source. Many sophisticated LIMS are designed with specific usage strategies in mind or are tuned to process specific vendor setups. As useful as they may be for larger operations, such LIMS customarily require significant attendance to set them up, to run and to maintain them—even to the extent that they are prohibitively demanding when it comes to deploy them for less complex tasks. Although most probably all of the available LIM systems might already be able to include / add a sample or project tracking function their costs will be to high for many smaller core facilities on tight budgets as most of them are commercial. Even those core facilities, which already have a working LIMS in place benefit from TraqBio, as it is easier and less tedious to add the tracking functionality by using TraqBio, than by adapting the LIMS.

In contrast, in a highly flexible, research-driven environment of genomic, proteomic, bioinformatics or other service units, complex management of internal multi-user projects is often of a lesser concern. On the contrary, efficient communication about project status is rather important. This can be realized via TraqBio. Moreover, the inclusion of a high number of different possible workflows within one core unit by such a tracking and communication tool requires a high level of flexibility to integrate a wide spectrum of approaches. In TraqBio this requirement is met by making use of a simple modular concept, which is intuitively comprehensible and does not require extensive training. TraqBio can readily be installed as stand-alone web application or integrated into an existing web server setup.

## Project Management

**Fig 2:**
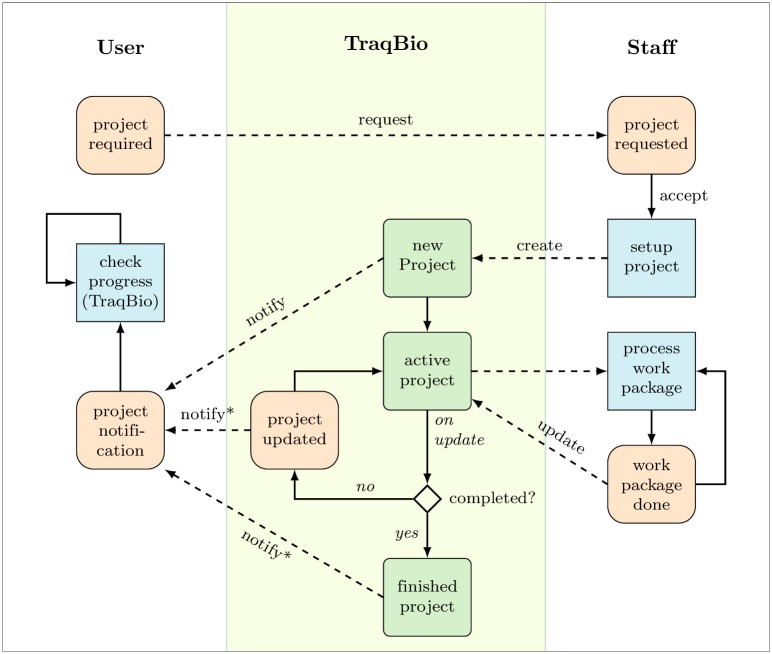
Workflow of a TraqBio project from the project request until the project is finished. The notifications marked with * are optional and can be switched off in the TraqBio configuration.

TraqBio uses a simple, modular project concept which represents a project as a sequence of work packages. Each project carries an identifier (name), comes with a description, and is assigned to a responsible person (advisor) of the core unit. Within a project each step, *i.e.* work package, can exist in two mutually exclusive stages: pending or done. A free text property allows to provide additional information about the work package or its outcome (result) to the TraqBio user. The name and the state of a work package are mandatory whereas the other properties are optional. A project is finished when all its work packages have been completed. The workflow of a TraqBio project is shown in Fig 2. A TraqBio project contains at least the names and e-mail addresses of the associated users. Additional information, such as the advisor and a description, as well as file upload capabilities, *e.g.* for order forms or a sample sheet can be provided as part of a project module. Core unit members, expediting the work packages of a project, update the status of individual work packages via their staff account. The user, however, does not need a TraqBio account. Tracking links are provided by the core unit advisor upon project commencement and enable users access to the tracking page.

In the following, the modular functionality of TraqBio is presented in general terms. The core unit user requests a new project without any interaction with TraqBio by approaching the core unit staff. Once the design of the project has been finalised by direct contact of the user with the core unit, the core unit staff (advisor) creates this new project in TraqBio. This triggers TraqBio to send a notification by e-mail to the user containing the tracking link of the project for accessing the progress page of that project. From now on, the user may at any time inform oneself about the progress of the project. As the project advances the core staff (advisor) updates the progress information through his administrative access to TraqBio. This information is becoming available immediately to the user upon accessing the project progress page. Additional information related to the execution or the results of a work package may be communicated to the users via the free text property for any of the work packages. Optionally, users and selected core staff may be notified about completed work packages via e-mail. This will be useful in situations, where e.g. the user intends to analyse intermediate results or in cases where the project requires attendance by (additional) core staff other than the one assigned to the project at its outset. Once all work packages are completed, the project is concluded by moving it to an archive. At the same time, results of the project are transmitted to the user by means agreed upon between the core unit and the user at the initialisation step of the project. Although TraqBio does not contain the functionality to store and transfer results of a working step to the user, it is possible to provide a link to a download location using the free text field of that working step.

Even careful planning and set-up of a project will not prevent the necessity to, possibly on the basis of intermediate results, modify the chain of work packages in a project by modification of the initial set-up in TraqBio. If such a situation arises, work packages could be deleted from or added to the initial scheme by the core unit staff without having to redesign the entire TraqBio flow for a project. It might also be necessary over the time to modify workflow templates, i.e. due to the inclusion of an additional quality control step. This flexibility and adaptability for *ad hoc* modifications is built into TraqBio to reflect and to respond appropriately to the real life situations encountered in a typical research environment.

TraqBio accommodates the definition of project templates for standard projects containing identical work packages. These can also be used as a starting framework for creating more complex projects. Such templates carry a specific identifier.

When creating a new project the core unit staff may choose to create a template in addition to the specific project at hand. The template contains the same set of work packages as the project. Still, such templates may also be created and modified independently in preparation for requests of repetitive routines expected within the framework of a screening project.

TraqBio comes with a *timeline view* (Fig 1C) providing an overview of actions taken by the core unit staff, including the creation or modification of projects. This allows for an instantaneous reviewing of changes made to projects over time including the date of the change and the responsible core unit member. Error messages, e.g. for invalid e-mail addresses, are displayed in the timeline as well.

Incidentally, the TraqBio functionality described above could also be used quite general in a research lab environment, where the individual researcher would manage a project (akin to the staff of a core unit) and the PI could take the role of the user to monitor projects at any time. This application of TraqBio might be particularly helpful in larger research groups or collaborative network projects run by several groups.

## Application Scenarios

Below, we illustrate the functionality and adaptability of TraqBio for three wide-spread types of core facilities, *i.e.* Proteomics, Genomics and Bioinformatics units. We also show, that TraqBio is useful for co-ordination by tracking projects, which require the services of two core units.

**Core Facility Proteomics.** Mass spectrometry and proteomics facilities provide access for members of the research institution to state-of-the-art proteomics technology. The available instrumentation and protocols allow for qualitative and quantitative characterization of protein-containing samples irrespective of their degree of complexity. As an example, it is possible to analyse samples containing single purified proteins to assess purity and identity [3] or intermediate complexity samples in the course of protein interaction studies [4], but also to provide data on highly complex samples such as whole cell lysates [5].

While this is an optional step in samples of low complexity, medium to highly complex samples often necessitate the use of fractionation by means such as SDS-PAGE or strong cation exchange (SCX) chromatography prior to mass spectrometric analysis. In addition, most proteomic experiments involve quantitative tasks using a diverse set of techniques such as label-free analysis, stable isotope labelling in cell culture (SILAC) or isobaric tagging. Depending on the complexity of the experiment and the question underlying the research involved, various bioinformatical steps and statistics have to be used before the data can be handed to the user of the core facility. In order to accommodate this multitude of possible experimental setups in proteome research, a tracking system has to be highly flexible, yet still has to be lean for the user to be able to intuitively see the progress on his project. TraqBio focusses on the presentation of a simple overview of the current project status to the core unit user, while still having the aforementioned flexibility to address multiple possible experimental designs. With the abdication of a user management, including passwords, TraqBio provides a low barrier to core unit users to actually retrieve their project status, thus reducing the number of contacts with staff to discuss project progression.

As an example of a more slender proteomic experiment, the workflow for a typical project at this core unit, the qualitative assessment of the protein contained within a non-complex sample using a gel-free approach is given in Fig 1B. Following the initial request for analysis, samples are delivered to the proteomics unit by the user. At the core unit, a sample is prepared for processing, frequently by employing protein precipitation to remove buffer components which may interfere with later steps in the process. In the next work package, proteins are typically digested by using proteases. The resulting peptides are then analysed by mass spectrometry and the resulting fragmentation spectra are matched against a database to identify the corresponding peptide sequences. This leads to the identification of the protein’s identity in the sample delivered, which then is returned to the user to conclude the project. An alternative, more complex workflow, employing a phosphoproteomic experiment, is given in Fig A in S1 File. Both workflows demonstrate the flexibility and versatility of TraqBio to accommodate the requirements of tracking tools faced at proteomic facilities.

**Fig 3:**
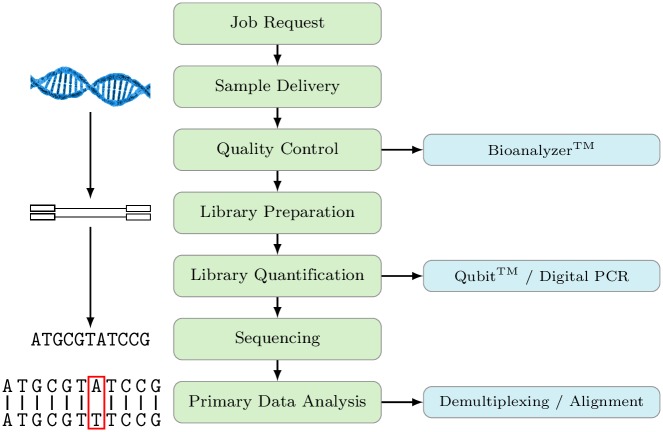
Example of a typical workflow in Next Generation Sequencing projects.

**Core Facility Genomics.** The genomics core facility described here provides state-of-the-art genomics equipment to researchers inside and outside of the research unit and also provides assistance with experimental design, trouble shooting, and data analysis. As an example, next generation sequencing services are offered ranging from whole genome to ChIP-Seq together with microarray analysis and production capabilities for self spotted microarrays. A typical workflow for a Next Generation Sequencing Project [6] is given in Fig 3. Such projects can be time consuming and may run over several weeks. Following quality control of the samples (DNA/RNA), so-called libraries are generated which, after quantification and normalization, are subsequently subjected to the actual sequencing step. That step may take some days in order to provide the intended coverage and statistics for the material submitted to the core unit. In addition, as it is imperative to minimise costs, it is necessary to run the high throughput instruments at maximum capacity with respect to the number of different samples processed at the same time in the sequencer. This entails the running of several projects in parallel combined in a single sequencing run. As the raw data generated by the sequencer are not readily interpretable for the user(s), they first need to be demultiplexed by assigning these individual reads to their respective samples/projects and in a second step align them to a reference genome. At that stage the sequencing data are transferred to and further processed by a bioinformatics unit as described below. In such a situation where the completion of a project involves two or more core units, it would even be more difficult for the user to keep track of the status of their project. However, as a TraqBio project is accessible and can be updated by all staff members involved, it is quite convenient to track the progress of a project in its entirety—even if several core units are involved. Fig B in S1 File gives another example of a workflow quite common in genomic core-facilities. Here, a microarray experiment for the detection of genomic or transcriptomic differences is depicted, which might also run for several weeks depending on the number of samples.

**Fig 4:**
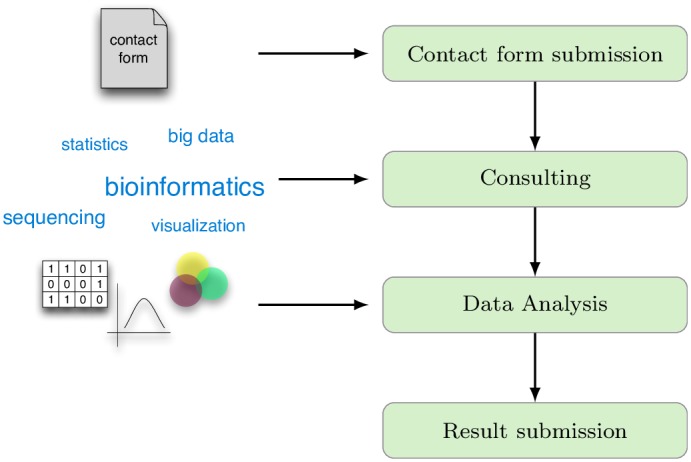
Typical workflow for a bioinformatics consulting service. After submission of a contact form, a meeting for the first consulting is scheduled. In this meeting, the researcher’s questions are discussed with the bioinformatics experts. After the data analysis has been carried out by the bioinformatics team, results are submitted to the researcher.

**Consulting—Bioinformatics Service Unit.** Core units for bioinformatics usually provide consulting services to researchers and may cover such diverse areas as biostatistics, sequencing analysis, data mining, and systems biology. A typical workflow for this consulting service is given in Fig 4. Researchers have to file a contact form before consulting, wherein basic information (*e.g.* research field, data type) and a short statement on the particulars of the requested service is provided by the user. A data analysis project is worked out in a first meeting between core staff and the user. After delivering data analysis results to the user, and discussion between user and core unit member(s), the project might be concluded or extended to address more in-depth questions, e.g. robustness analyses [7, 8] and classification experiments [9, 10] (see supplementary information for an alternative workflow, Fig C in S1 File).

The scenarios described here are only examples for possible usages of TraqBio. Due to the open source nature of the program other research groups are invited to adopt the program to other scenarios as well.

## Technical Considerations

TraqBio was implemented by using the Clojure programming language, which is a Lisp dialect running on the Java Virtual Machine [11]. The focus of Clojure on easy concurrent programming and the vast amount of libraries in the Java ecosystem are a substantial aid for the development of web applications like TraqBio. TraqBio may be run either as a stand-alone application using an embedded web server or integrated into state-of-the-art web servers. The stand-alone application requires only a simple setup involving three steps: (i) generation of a default configuration file using TraqBio, (ii) adjusting the configuration file with respect to individual requirements, and (iii) starting of TraqBio. A fully featured TraqBio application can quickly be set up in demo mode for evaluation purposes using the default configuration. Wherever it is required TraqBio can easily be integrated into an existing web server setup (*e.g.* Apache [12]).

Upon deployment as stand-alone application, it is recommended to set up secure communication (SSL, https) by enabling this feature during the TraqBio configuration step. When integrated into a web server either TraqBio or the web server itself may be set up to ensure secure communication. TraqBio stores information about failed login attempts in a log file such that intrusion attempts are recorded and may be prevented using established external tools. The tracking numbers used for the tracking links are randomly generated universal unique identifiers [13] that prevent guessing tracking links easily. Project data remain completely private within the core unit due to the installation of TraqBio on a server of the core unit. TraqBio uses SQLite as an embedded database which permits effortless relocation of a TraqBio setup and saves license costs for databases of commercial vendors.

TraqBio also exposes a RESTful [14] scripting API that allows to set up automated work packages. After the completion of a manual work package, the core staff attaches for instance the result data to the project as sample sheet. External programs run by the core unit staff are now able to query the scripting API automatically to find all projects whose active work package is processable and to download the corresponding sample sheets. After processing the sample sheets the work packages can be completed by the program using the scripting API. The additional information of the work package (e.g. free text) can also be updated at the same time. To use this API, the external program only needs to use a valid TraqBio staff account to login. Due to the RESTful API the external program is not required to be run on the TraqBio server. Any computer with a connection to the TraqBio server can be used. The scripting API is programming language agnostic so that any programming language can be used. The TraqBio repository also contains an example bash script that demonstrates the usage of this scripting API.

**Fig 5:**
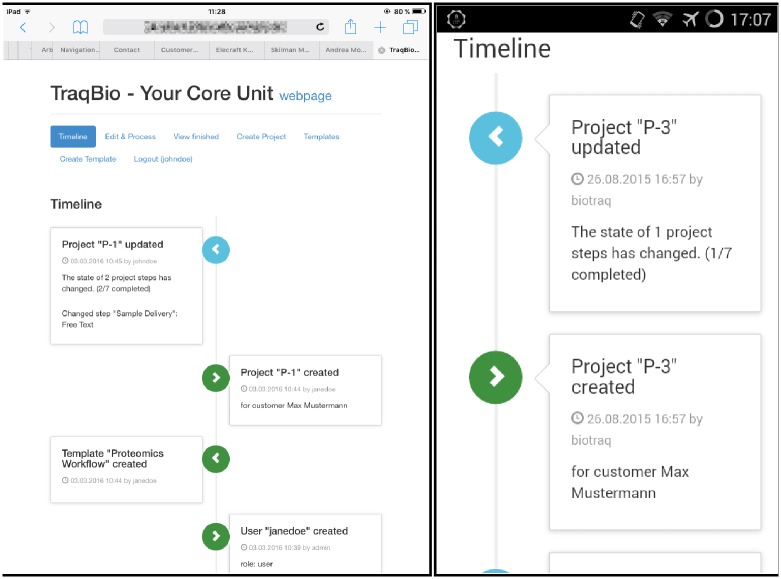
TraqBio is also well accessible on mobile devices like tablets (left screenshot) and smartphones (right screenshot) due to its responsive design.

By design, TraqBio adapts the layout of its GUI to different screen geometries (Fig 5). Therefore, TraqBio can be accessed without restrictions from the entire range of monitors found on classical desktop computers to tablets or even smartphones, thereby rendering it a versatile everyday tool.

## Discussion

The conceptually simple modularization of a project as implemented in TraqBio represents the organization and workflow of most core unit projects well enough and avoids elaborate customization that is often necessary if more complex models are evoked. While being a core feature of TraqBio, this simplicity might not cater to all variants of workflows encountered in e.g. genomics or proteomics. If, for example, the same sample is analysed in different workflows, the linear concept in TraqBio’s design approach does not allow such a branching of workflows directly, but by invoking two workflows which are then interrelated through information in the text fields. Nevertheless, the main purpose of TraqBio is to keep the user well informed about the status of requested services, and not bother the user with the requirement of an intricate workflow knowledge in those used technologies such as genomics or proteomics. For that purpose the sequential workflow model of TraqBio is ideal to deliver the desired information. Communicating progress information via TraqBio only requires that the core unit staff updates the project information only once per completed work package, thereby saving valuable time spent in frequent *ad hoc* communication, but at the same time still keeping the user informed. Additionally, TraqBio documents project workflows and the course of the individual projects.

The easy setup process of TraqBio allows a quick installation on the designated web server and enables a quick demo installation of a fully featured application for evaluation purposes. Following installation, TraqBio is immediately ready for use without any complex configuration process and does not require any additional extensive training of the core unit staff. Following a short test period, TraqBio is now successfully used in three core units at Ulm University.

## Supporting Information

S1 File Supplement containing additional workflow scenarios and user experiences. (PDF)
