# Supplementary material for: TraqBio - Flexible Progress Tracking for Core Unit Projects
Source: PLoS One. 2016 Sep 27;11(9):e0162857. doi: 10.1371/journal.pone.0162857 (PMC5038959; doi:10.1371/journal.pone.0162857)
Supplement: S1 File — (PDF) [file pone.0162857.s001.pdf]

Supplement to

TraqBio - Flexible progress tracking for core unit projects

Additional workflow scenarios and user experiences

Gunnar Völkel[1,☯], Sebastian Wiese[2,☯], Karlheinz Holzmann[3,☯], Johann M. Kraus[1,☯], Fabian Schneider[1], Matthias Görlach[4], Hans A. Kestler[1,4,*]

1 Institute of Medical Systems Biology, Ulm University, D-89069 Ulm, Germany
2 Core Unit Mass Spectrometry and Proteomics, Ulm University, D-89069 Ulm, Germany
3 Core Facility Genomics, Ulm University, D-89069 Ulm, Germany
4 Leibniz Institute on Aging – Fritz Lipmann Institute and FSU Jena, D-07745 Jena, Germany

☯These authors contributed equally to this work.
* Corresponding Author: hans.kestler@uni-ulm.de

This supplement contains additional workflow scenarios for the proteomics and genomics core units to showcase the applicability and flexibility of TraqBio. Additionally, the consulting projects of the bioinformatics service unit are described more detailed. The supplement finishes with feedback provided by the proteomics and genomics core units.

Core Facility Proteomics

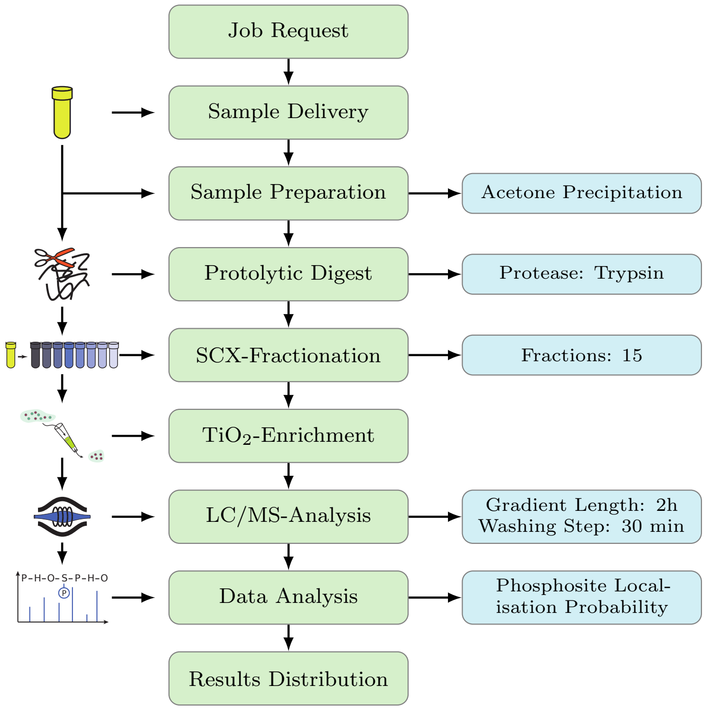

Figure A: Workflow of a phosphoproteomic experiment. Two steps, SCX fractionation and TiO2-enrichment of phosphopetides, are added to the gel-free workflow (main paper Figure 2B) to seperate and specifically enrich phosphopeptides prior to LC/MS-analysis.

Core Facilitiy Genomics

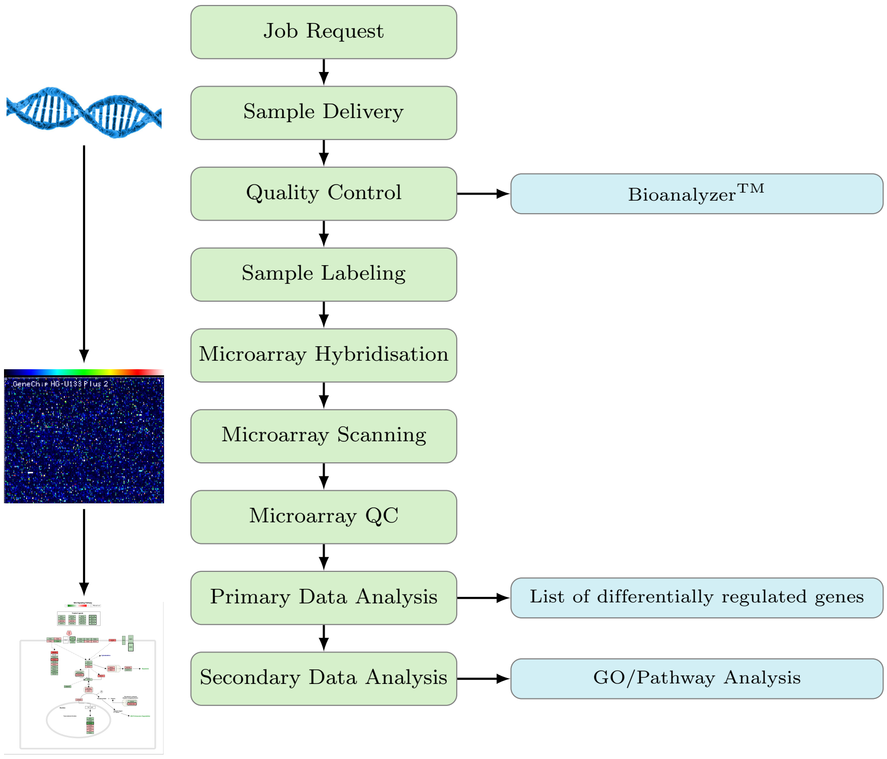

Figure B: Example of a typical workflow of a microarray experiment as it is typically carried out in academic core facilities.

Consulting – Bioinformatics Service Unit

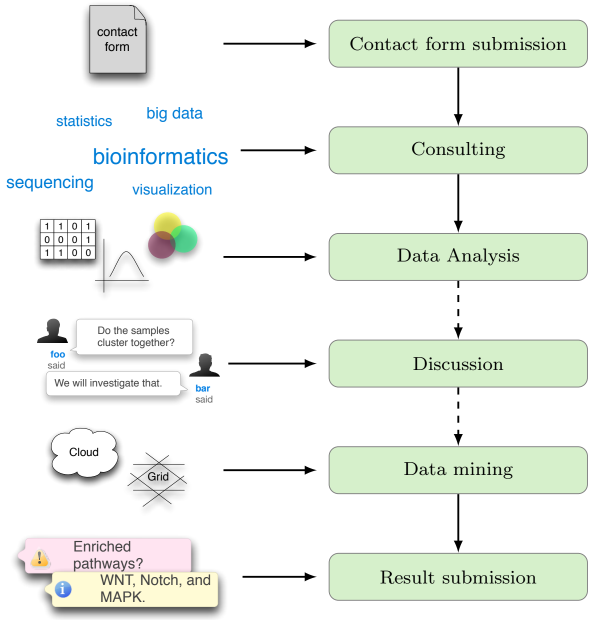

Figure C: Typical workflow for a bioinformatics consulting service. After submission of a contact form, a meeting for the first consulting is scheduled. In this meeting, the researcher's questions are discussed with the bioinformatics experts. The appointment for data analysis may include descriptive statistics and hypothesis testing. After the data analysis has been calculated by the bioinformatics team, results are submitted to the researcher. Optionally (dashed line), another meeting for discussion of results can be scheduled. In this meeting, further data mining may be requested by the researcher. This may include the application of data mining techniques (e.g. robustness analysis [1, 2], classification [3]) by involving cloud computing and/or grid compute servers [4]. The flexibility of TraqBio allows for the implementation of any required modification of such a stepwise project extension to keep the user informed.

User experiences

From the Genomics Core Unit: We received very positive feedback from our customers. All emphasized the ease of use, so that frequent intermediate requests on the status of a project nearly dropped to zero. The only past request for an additional functionality is now included in the current version. In the beginning users had to actively check the system for the status of their projects and so the request was made to automatically notify the customers upon completion of a particular step in the worflow. Other than that no further requests have been made by our customers.

From the Proteomics Core Facility: We received overall positive feedback from our users. Using TraqBio reduced status update requests by users significantly. An additional functionality request by staff members from our core unit was addressed. This concerned the ability to save templates not only at project inception, but also over the course of the experiments after possible additional information had been added. This request was addressed early on and is now a feature of TraqBio.

In addition, the Leibniz Institute on Aging (Fritz Lipmann Institute) in Jena (Germany) has organized its technology platforms into Core Facilities and Services as of January 1st 2016 (see: http://www.leibniz-fli.de/research/core-facilities-services/) ranging from Proteomics and DNA Sequencing facilities to Histology and Media Services. TraqBio is currently being setup in these facilities.

References

[1] Kraus JM, Kestler HA. A highly efficient multi-core algorithm for clustering extremely large datasets. BMC Bioinformatics. 2010;11(1):169+.

[2] Kraus JM, Müssel C, Palm G, Kestler HA. Multi-objective selection for collecting cluster alternatives. Computational Statistics. 2011;26(2):341–353.

[3] Müssel C, Lausser L, Maucher M, Kestler HA. Multi-Objective Parameter Selection for Classifiers. Journal of Statistical Software. 2012;46(5):1–27.

[4] Völkel G, Lausser L, Schmid F, Kraus JM, Kestler HA. Sputnik: ad hoc distributed computation. Bioinformatics. 2015;31(8):1298–1301.
